# Endothelial Transcytosis of Lipoproteins in Atherosclerosis

**DOI:** 10.3389/fcvm.2018.00130

**Published:** 2018-09-25

**Authors:** Xinbo Zhang, William C. Sessa, Carlos Fernández-Hernando

**Affiliations:** ^1^Vascular Biology and Therapeutics Program, Integrative Cell Signaling and Neurobiology of Metabolism Program, Department of Comparative Medicine and Department of Pathology, Yale University School of Medicine, New Haven, CT, United States; ^2^Vascular Biology and Therapeutics Program, Department of Pharmacology, Yale University School of Medicine, New Haven, CT, United States

**Keywords:** transcytosis, lipoprotein, endothelial cell, atherosclerosis, LDL, HDL

## Abstract

Seminal studies from Nikolai Anichckov identified the accumulation of cholesterol in the arteries as the initial event that lead to the formation of atherosclerotic plaques. Further studies by Gofman and colleagues demonstrated that high levels of circulating low-density lipoprotein cholesterol (LDL-C) was responsible for the accelerated atherosclerosis observed in humans. These findings were confirmed by numerous epidemiological studies which identified elevated LDL-C levels as a major risk factor for cardiovascular disease. LDL infiltrates in the arterial wall and interacts with the proteoglycan matrix promoting the retention and modification of LDL to a toxic form, which results in endothelial cell (EC) activation and vascular inflammation. Despite the relevance of LDL transport across the endothelium during atherogenesis, the molecular mechanism that control this process is still not fully understood. A number of studies have recently demonstrated that low density lipoprotein (LDL) transcytosis across the endothelium is dependent on the function of caveolae, scavenger receptor B1 (SR-B1), activin receptor-like kinase 1 (ALK1), and LDL receptor (LDLR), whereas high-density lipoproteins (HDL) and its major protein component apolipoprotein AI transcytose ECs through SR-B1, ATP-Binding cassette transporter A1 (ABCA1) and ABCG1. In this review article, we briefly summarize the function of the EC barrier in regulating lipoprotein transport, and its relevance during the progression of atherosclerosis. A better understanding of the mechanisms that mediate lipoprotein transcytosis across ECs will help to develop therapies targeting the early events of atherosclerosis and thus exert potential benefits for treating atherosclerotic vascular disease.

## Introduction

Atherosclerosis is a chronic inflammatory process involving complex interactions of normal and modified lipoproteins, monocytes, macrophage-foam cells, T lymphocytes, endothelial cells (ECs), smooth muscle cells, and fibroblasts. The transendothelial transport of apoB-lipoproteins plays a pivotal role in the pathogenesis of atherosclerosis. According to the “infiltration theory,” the development of atherosclerosis is triggered by the entry and subendothelial retention of lipoprotein from the bloodstream, particularly low density lipoprotein (LDL), and apolipoprotein-B (apoB)-containing remnants within the arterial wall (1). HDL must also cross the endothelial barrier in the arterial wall to exert its athero-protective properties mediating cholesterol efflux from lipid laden macrophages. Increased cholesterol influx relative to efflux through ECs and enhanced binding to extracellular matrix result in the retention of both pro-atherogenic (apoB-lipoproteins) and anti-atherogenic lipoproteins (HDL) in atherosclerotic arteries (2). Although, lipoprotein transport is a critical for the initiation and progression of atherosclerosis, the fluxes of lipoproteins into and out of the artery wall have not been completely investigated and the mechanisms by which LDL and HDL enters into the subendothelial space remains unclear.

## Transcytosis in EC

Arteries and veins are composed of various layers of smooth muscle cells, connective tissue, and a thin single sheet of ECs. The endothelium forms a barrier due to the presence of specialized cell-to-cell junctions which selectively regulate the passage of molecules and cells between the bloodstream and tissues by the paracellular route (3). The EC barrier is involved in many systemic processes including vascular tone, fluid homeostasis and host defense. The endothelium is permeable to water and small molecules with a diameter below 6 nm, but nearly impermeable to macromolecules with different endothelia endowed with unique “perm-selectivity” (4). The transport of macromolecules, including lipoproteins, across the endothelium is actively controlled by ECs via the transcellular route, or transcytosis (5, 6). The process of transcytosis involves fluid phase or receptor-mediated ligand uptake by endocytosis, transition of the cargo through the cytoplasm, and exocytic release of the cargo (7).

The transcytosis can be separated into indirect and direct pathways dependent on their transportation routes. In the case of the indirect transport pathway, molecules are endocytosed into early endosomes, transferred to recycling endosomes, and exocytosed on the opposite side of EC layer (8). The indirect transcytosis routes are also receptor-specific, and rely on the interaction of molecules and their endogenous receptors. For example, LDL can transcytose across the EC layer by binding to LDL receptor (LDLR) or activin-like kinase 1 (ALK1) protein, which will be described in more detail in section Transcytosis of LDL in EC of this review article (9, 10). The direct transcytosis route involves the direct transport of the molecules from the incoming side to the opposite side, which in turn fuse with the basolateral aspects of the plasma membrane (8). Caveolae-mediated transport in endothelia is the most common route of direct transcytosis. Besides the receptor-specific indirect transcytosis and direct transcytosis routes, proteins or other macromolecules can be transported via nonspecifically binding to membranes through electrostatic interaction and fluid-phase transcytosis (11).

## Transcytosis of LDL in EC

**Figure 1:**
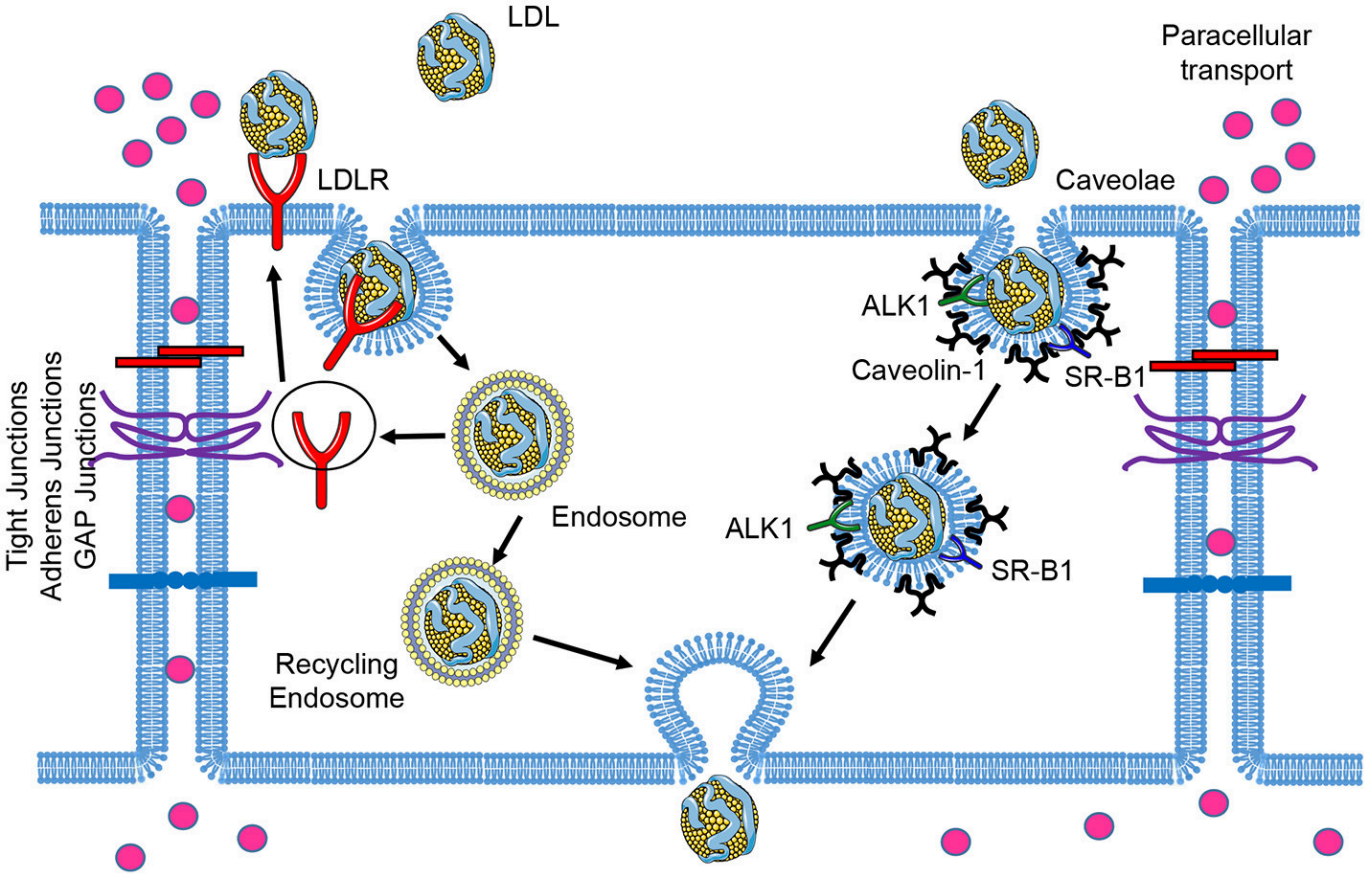
Schematic of low-density lipoprotein (LDL) transcytosis by ECs. Water and small molecules with a diameter below 6 nm are transported across endothelial cells by the paracellular route. The classical LDL receptor (LDLR) pathway mediates LDL uptake and its degradation in the lysosomes, which is not essential for transcytosis. LDL can transpose ECs through receptor-mediated transcytosis associated with scavenger receptor B1 (SR-B1), activin receptor-like kinase 1 (ALK1), or LDL receptor (LDLR, only occurred in brain ECs), as well as caveolae-mediated direct transcytosis. The endocytosed LDL particles are then transferred to the opposite side of the cell directly (caveolae-mediated transcytosis) or indirectly (receptor-mediated transcytosis) and exocytosed to subendothelial space.

LDL particles contain one single apolipoprotein B-100 (apoB-100) molecule and carry the majority of the cholesterol in circulation. Elevated circulating LDL levels are highly related to the development of atherosclerosis and coronary heart disease. Mutations of LDLR cause an autosomal dominant disorder, familial hypercholesterolemia (FH), which is characterized by elevated plasma concentrations of LDL cholesterol and early coronary heart disease (12). During the initial stages of atherosclerosis, LDL particles are transported across the EC barrier and accumulate in the subendothelial space. The trapped LDL molecules are oxidized to form oxidized LDL (oxLDL), which facilitates the uptake of these modified lipoproteins by the scavenger receptors expressed in macrophages. This promotes the formation of macrophage foam cells within atherosclerotic lesions (13). Early electron microscopy studies examining LDL transport across the endothelium documented the uptake of LDL via two routes: a saturable, clathrin-mediated endocytic mechanism via LDLR; and fluid-phase non-saturable transcytotic mechanism through non-coated plasmalemmal vesicles, perhaps caveolae (14). In this section, we will review the receptors that have been associated with the transport of LDL across the endothelium and the relevance of caveolae in this process (Figure 1).

### Endothelial LDLR-mediated LDL transcytosis

The endocytosis of LDL by LDLR has been extensively characterized (15). ApoB in LDL particles is recognized by LDLR, promoting LDL uptake into the cells. The internalized particles are routed to endosomes, where LDL dissociates from LDLR because of the low PH in endosomal lumen. Dissociated LDL is transferred to late endosomes and lysosomes for further degradation, whereas LDLR is recycled back to the plasma membrane. Besides to its function in LDL for utilization and clearance for peripheral tissues, LDLR has been reported to mediate LDL transcytosis in the blood-brain barrier (BBB) (16). An antibody known to interact with the LDLR-binding domain inhibited the transcytosis of LDL, and the LDL transcytosis paralleled the increase in LDLR expression. This suggests that LDL is transcytosed by a receptor-mediated mechanism (16). The non-degradation of the LDL during transcytosis indicates that the transcytotic pathway in brain capillary endothelial cells is different from the classical LDL receptor pathway of endocytosis (16). However, this is unlikely to be the case in ECs of systemic circulation, whereas PCSK9-mediated LDLR degradation has no effect on LDL transcytosis (17), indicating that the transcytosis process in ECs of systemic circulation is LDLR-independent.

### Scavenger receptor B1 (SR-B1) and LDL transcytosis in EC

SR-B1 is well-known as high affinity HDL receptor which mediates the selective uptake of HDL cholesterol ester into the liver and the bidirectional flux of free cholesterol between cells and HDL (18, 19). Recent studies have revealed an unexpected role of SR-B1 in regulating LDL transcytosis in ECs (9, 17). Lee et al. using a novel total internal reflection fluorescence (TIRF) microscopy approach, demonstrated that SR-B1 silencing significantly attenuates LDL transcytosis in human coronary ECs (17). These results were further supported *in vivo* by assessing the accumulation of fluorescence-labeled LDL (DiI-LDL) in aortas isolated from WT and SR-B1 deficient mice. The authors found a significant reduction in LDL infiltration into the subendothelial space in aortas from SR-B1-deficient mice perfused *ex vivo* with DiI-LDL (17). Similar findings were observed by Sessa and collaborators, who identified SR-B1 as a relevant receptor that controls LDL uptake in ECs using a genome-wide RNAi screening strategy (9). Mechanistically, it is not clear how SR-B1 mediates LDL transport since there is no data showing that it directly binds apoB on LDL. However, it is feasible that SR-B1 is part of a complex that facilitates LDL uptake through additional pathways. Specific expression of SR-B1 in endothelial cells showed 37% less aortic lesions compared to control mice which was attributed to decreased plasma cholesterol and increased HDL levels (20), however, the contribution of SR-B1 in lipoprotein transcytosis on the development of atherosclerosis is still unclear.

### ALK1-mediated LDL transcytosis in EC

In addition to SR-B1, we have identified ALK1 as a novel low-affinity, high-capacity receptor for LDL in EA.hy926 cells, an immortalized human endothelial-like cell (9). ALK1 is an EC-restricted TGF-β-type 1 receptor with high affinity to the bone morphogenetic protein (BMP) 9 and 10 ligands (21). By knockdown or overexpression studies, ALK1 was found to mediate LDL transcytosis independent of its kinase activity and that ALK1 can directly bind LDL (9). Moreover, the specific deletion of ALK1 in the endothelium significantly reduced DiI-LDL uptake into the aortic endothelium by *en face* confocal imaging of isolated vessels (9). Interestingly, ALK1-dependent uptake of LDL does not result in its lysosomal degradation, implying a distinct internalization pathway from LDLR (9).

### Caveolae regulation of LDL transcytosis across the endothelium

Caveolae are small bulb-shaped plasma membrane invaginations present in most cell types with ~50–80 nm in diameter (22). Caveolin-1 (Cav-1) is the major structural protein essential to the formation of the caveolae in endothelial cells (23), which have been implicated in various physiological and pathological contexts based on their cellular functions in lipid homeostasis, signal transduction and endocytosis (24–26). We and others have demonstrated that the absence of Cav-1 protects mice against the progression of atherosclerosis (25, 27, 28). Importantly, re-expression of Cav-1 in ECs attenuates this effect and promotes lesion expansion (25). Mechanistically, we demonstrated that genetic ablation of Cav-1 significantly impairs LDL transport and retention in the arterial wall (25). Similar findings were observed by Lisanti and colleagues, who found a significant reduction in radioactively-labeled LDL accumulation in mice lacking Cav-1 (29). These findings suggest that caveolae plays a relevant role as a major regulator of LDL entry into the vessel wall and participates initiation of atherosclerosis. Moreover these findings reinforce the original observations identifying non-coated plasmalemmal vesicles as caveolae, and the major entry pathway for with LDL (14). Importantly, SR-B1 and ALK1 are located in caveolae, suggesting that both receptors might promote specific LDL binding and loading to caveolae facilitating the transport of LDL across the endothelium (30, 31). Additional experiments will be important to dissect the specific contribution of caveolae in SR-B1 and ALK1-mediated LDL transcytosis.

## Transcytosis of HDL in EC

### SR-B1- and ABC transporters- mediated HDL transcytosis in EC

The protective effect of HDL is attributed to its ability in mediating reverse cholesterol transport (RCT) through which cholesterol is delivered from the periphery (such as arterial wall cells including lipid laden macrophages) back to the liver for biliary excretion (32, 33). To achieve the removal of excess cholesterol deposited in the atherosclerotic lesions, HDL must first cross the endothelial barrier to get access to macrophage foam cells in atherosclerotic plaques. The transcytosis of apolipoprotein A-I (apoAI), the major protein constituent of HDL, from the apical to the basolateral compartment was observed in ECs with minor amounts of the internalized apoAI degraded (34). The mechanism of apoAI transcytosis through ECs were further confirmed by the same group, who showed that apoAI is colocalized with ATP-Binding cassette transporter A1 (ABCA1) and that pharmacological intervention or RNA interference of ABCA1, but not SR-B1, modulated the transcytosis of apoAI through ECs (35). However, the endothelial transcytosis of mature HDL is different from that of apoAI, as it is dependent on SR-B1 and ABCG1 but not ABCA1 (36). Interestingly, the transcytosis of LDL mediated by SR-B1 appears to be regulated by (VEGF)-A, linking vascular permeability with enhance LDL transcytosis via SR-B1 (37). In addition, the transcytosis of HDL in brain microvascular ECs was demonstrated to be partially SR-B1-dependent and inhibition of Cav-1, or clathrin and adaptor protein PDZ Domain Containing 1 (PDZK1) had no impact on the HDL transcytosis (38).

Delivery of excess cholesterol from peripheral tissues and cells to the bloodstream by HDL is the initial steps in RCT process (32, 33). The lymphatic system is considered to be the primary location for the return of lipoproteins from the interstitial space to circulation (39). It has been shown that lymphatic transport of cholesterol by HDL is mediated via SR-B1 expressed on lymphatic endothelium using silencing RNA interference, neutralizing antibody, and transgenic mice (40–42). The specific function of SR-B1 is dependent on the uptake and transcytosis of HDL in lymphatic endothelial cells (40–42). Importantly, SR-B1 was found to be present in both the basolateral and apical membranes of ECs, but greater cellular uptake of cholesterol from HDL was found in the basolateral compartment (20). Enhanced expression of SR-B1 in ECs resulted in decreased atherosclerosis, supporting a possible role for endothelial SR-B1 in the flux of cholesterol across ECs (20).

### Caveolae-mediated HDL transcytosis in ECs

Immunohistochemical studies have shown a partial co-localization of DiI-labeled HDL in caveolae and gold-labeled HDL with Cav-1, but the potential role of Cav-1/caveolae on HDL trafficking in ECs remains poorly understood (43). Caveolae have been also implicated in the regulation of cellular cholesterol homeostasis and a number of cholesterol-trafficking steps (44–46). The reconstitution of purified Cav-1 only with cholesterol-containing lipid vesicles revealed the first direct link between Cav-1 and cholesterol (47). A series of studies by Fielding and Fielding suggest that caveolae may act as portals for cholesterol efflux upon incubation of cells with HDL (44, 48, 49). These data was further supported by *adenovirus-mediated* overexpression of Cav-1 in the mouse liver, caused an increase in plasma HDL-cholesterol (45, 50, 51). Cav-1/caveolae may also regulate the activity of scavenger receptors and ATP-binding cassette transporters (ABC)s, that control the cholesterol homeostasis in ECs. While these studies suggest an important role for caveolae in regulating HDL transport, cholesterol efflux, and hepatic HDL biogenesis, a recent study showed that HDL internalized by ECs did not colocalize with clathrin or Cav-1 and is independent of fluid phase uptake (52). Instead, HDL appeared to be internalized and trafficked by ECs through a non-classical endocytic route involving dynamin and cytoskeletal networks (52). The mechanisms of caveolae and Cav-1 involved in HDL transcytosis in ECs require further study.

## Conclusions

The subendothelial accumulation of pro-atherogenic lipoproteins including LDL represents a pivotal step in the initiation of atherosclerosis (53). On the contrary, removal of cholesterol from the subendothelial space to the circulation by HDL-mediated reverse cholesterol transport represents a relevant anti-atherogenic pathway (53). Endothelial transcytosis is considered to be a process that involves endocytosis, vehicle traffic through the cytoplasm and exocytic release of the cargo. So far, most of the studies on endothelial transcytosis have focused on the endocytosis step. The LDL transcytosis from the apical to the basolateral compartment in ECs is dependent on the function of caveolae, SR-B1, or ALK1 (Figure 1). ECs are quite heterogeneous depending on the tissue bed (54). LDLR-mediated LDL transcytosis only occurred in brain ECs, but not in ECs from system circulation. Unlike LDL, the transcytosis of HDL across ECs has been less investigated. SR-B1 is suggested to be involved in the dual transport of HDL between the bloodstream and peripheral tissues, whereas the role of other molecules including caveolae, ABCA1 and ABCG1 need to be further investigated.

The investigation of endothelial transcytosis of lipoproteins is hampered by limitations in our ability to observe or monitor the transcytosis process. The technological advances of an *in vitro* assay for endothelial transcytosis, by the continuing availability of super-resolution microscopy and live-cell imaging techniques, will help facilitate the delineation of the mechanisms and molecular regulation of endothelial transcytosis (17, 38). In addition, most of the present findings were investigated by *in vitro* assays, the observation of lipoprotein transcytosis through ECs *in vivo* are needed to prove the physiological relevance. Furthermore, the internalization step in endothelial lipoprotein transcytosis has been relatively well investigated, however, the regulation of intracellular vehicle traffic and exocytosis on the other side of cells remains poorly understood. The question of whether the composition of lipoproteins is altered during the process of endothelial transcytosis needs to be answered.

Numerous drug carriers targeting endothelial transcytosis, such as the caveolae-dependent pathway, have been developed for the treatment of cancer and lung injury patients (55). However, the precise control of lipoprotein transcytosis in ECs requires deeper understanding of the mechanisms and regulations involved in this process. For example, the dual role of SR-B1 in both LDL and HDL transcytosis raises a question of how to balance these different pathways to protect against atherogenesis. A better understanding of the mechanisms that mediate lipoprotein transcytosis across ECs may help to develop therapies targeting on the early events of atherosclerosis and thus exert potential benefits to the treatment of atherosclerotic vascular disease.

## Author contributions

XZ, WCS, and CF-H drafted, edited, and approved the manuscript and figures.

### Conflict of interest statement

The authors declare that the research was conducted in the absence of any commercial or financial relationships that could be construed as a potential conflict of interest.
